# Strategic considerations for invasive species managers in the utilization of environmental DNA (eDNA): steps for incorporating this powerful surveillance tool

**DOI:** 10.3391/mbi.2021.12.3.15

**Published:** 2021-07-09

**Authors:** Jeffrey Morisette, Stanley Burgiel, Kelsey Brantley, Wesley M. Daniel, John Darling, Jeanette Davis, Thomas Franklin, Keith Gaddis, Margaret Hunter, Richard Lance, Tracy Leskey, Yale Passamaneck, Antoinette Piaggio, Brian Rector, Adam Sepulveda, Melissa Smith, Carol A. Stepien, Taylor Wilcox

**Affiliations:** 1 National Invasive Species Council Staff, Department of the Interior, Office of the Secretary, USA; 2 U.S. Geological Survey Wetland and Aquatic Research Center, 7920 NW 71^st^ St. Gainesville, FL 32653, USA; 3 Center for Environmental Monitoring & Modeling, United States Environmental Protection Agency, Research Triangle Park, NC 27711, USA; 4 National Oceanic and Atmospheric Administration, 1401 Constitution Ave. NW, Floor 5, #58012RA, USA; 5 US Department of Agriculture Forest Service, National Genomics Center for Wildlife and Fish Conservation, Rocky Mountain Research Station, 800 E Beckwith Ave, Missoula, MT 59801, USA; 6 NASA Headquarters 300 E Street SW Washington, DC 20546, USA; 7 U.S. Army Corp of Engineers, CEERD-EPP, 3909 Halls Ferry Road, Vicksburg, MS 39180, USA; 8 USDA, Agriculture Research Service, Appalachian Fruit Research Station, 2217 Wiltshire Road, Kearneysville, WV 25430, USA; 9 U.S. Bureau of Reclamation, Ecological Research Laboratory, PO Box 25007 (86-68560), Denver, CO 80225, USA; 10 USDA, National Wildlife Research Center, Room A 116, 4101 La Porte Ave, Fort Collins, CO 80521, USA; 11 USDA, Agricultural Research Service, Great Basin Rangelands Research Unit, 920 Valley Road, Reno, NV 89512, USA; 12 U.S. Geological Survey, Northern Rocky Mountain Science Center, 2327 University Way Suite 2, Bozeman, MT 59715, USA; 13 USDA, Agricultural Research Service, Invasive Plant Research Laboratory, 3225 College Avenue, Fort Lauderdale, FL 33314, USA; 14 University of Washington and NOAA Pacific Marine Environmental Lab, Seattle, WA 98115, USA; 15 US Department of Agriculture Forest Service, National Genomics Center for Wildlife and Fish Conservation, Rocky Mountain Research Station, 800 E Beckwith Ave, Missoula, MT 59801, USA

**Keywords:** early detection, conservation genetics, National Invasive Species Council

## Abstract

Invasive species surveillance programs can utilize environmental DNA sampling and analysis to provide information on the presence of invasive species. Wider utilization of eDNA techniques for invasive species surveillance may be warranted. This paper covers topics directed towards invasive species managers and eDNA practitioners working at the intersection of eDNA techniques and invasive species surveillance. It provides background information on the utility of eDNA for invasive species management and points to various examples of its use across federal and international programs. It provides information on 1) why an invasive species manager should consider using eDNA, 2) deciding if eDNA can help with the manager’s surveillance needs, 3) important components to operational implementation, and 4) a high-level overview of the technical steps necessary for eDNA analysis. The goal of this paper is to assist invasive species managers in deciding if, when, and how to use eDNA for surveillance. If eDNA use is elected, the paper provides guidance on steps to ensure a clear understanding of the strengths and limitation of the methods and how results can be best utilized in the context of invasive species surveillance.

## I. Introduction

Over the past decade, the application and development of molecular techniques in the ecological community have rapidly expanded (Real 2017). Environmental DNA (eDNA) is broadly defined as any organismal DNA (e.g. microbial, macrobial, meiofaunal) present within a given environment (i.e., water, soil, sediment) (Pawlowski et al. 2020). In the domain of invasive species management, eDNA sampling and analysis (hereafter referred to as eDNA techniques) provides a highly sensitive approach to infer the presence of one or more targeted invasive species or the composition of multiple species in the community.

Every year in the United States (US), invasive species cause billions of dollars in economic losses and other damages (Executive Office of the President 2016). The federal government allocated nearly US $3 Billion to invasive species prevention and control efforts in fiscal year 2020, with similar investments in previous years (NISC 2020). A significant portion of that effort is dedicated to assessing the presence of invasive species, whether it is initial detection of an alien species of concern, tracking their spread, or monitoring for survivors of eradication efforts. Advances in molecular technologies provide significant power to detect evidence of a species’ presence in a given environment via its eDNA, even when its numbers are relatively low or the species is cryptic or otherwise difficult to differentiate morphologically from close relatives (Marshall and Stepien 2019; Martinez et al. 2020). In comparison with traditional collection and identification methods, eDNA techniques can be more sensitive, cost-effective, and targeted to the identification of species of interest; safer for wildlife and field staff; and less harmful to the ecosystem (Darling 2019). Consideration of wider utilization of eDNA techniques for invasive species surveillance is warranted. Yet, interpretation and use of eDNA results differ from the use and interpretation of traditional sampling. Also, since the field is rapidly evolving, its application may require considerable technical capacity.

In the US, the National Invasive Species Council (NISC) provides national leadership to coordinate, sustain, and expand federal invasive species management efforts. As part of its annual planning process, NISC identifies priority thematic areas that would benefit from inter-agency or intergovernmental collaboration. In the 2020 Work Plan, NISC selected eDNA techniques as one of six priority areas to highlight as an emerging tool for invasive species management across a range of federal agencies and actions (NISC 2019). The resulting article comes from the task team of nearly 30 federal scientists and invasive species specialists working at the nexus of eDNA techniques and invasive species management convened through the NISC eDNA techniques initiative.

While eDNA techniques are already in widespread use and hold promise as tools for invasive species detection and surveillance, there are several key questions to consider:

1. Why should an invasive species manager (referred to as managers herein) consider using eDNA?
2. Can eDNA help with your invasive species surveillance needs?
3. What are the important components to operational implementation?
4. How do you go about utilizing eDNA tools for surveillance?

**Figure 1.**
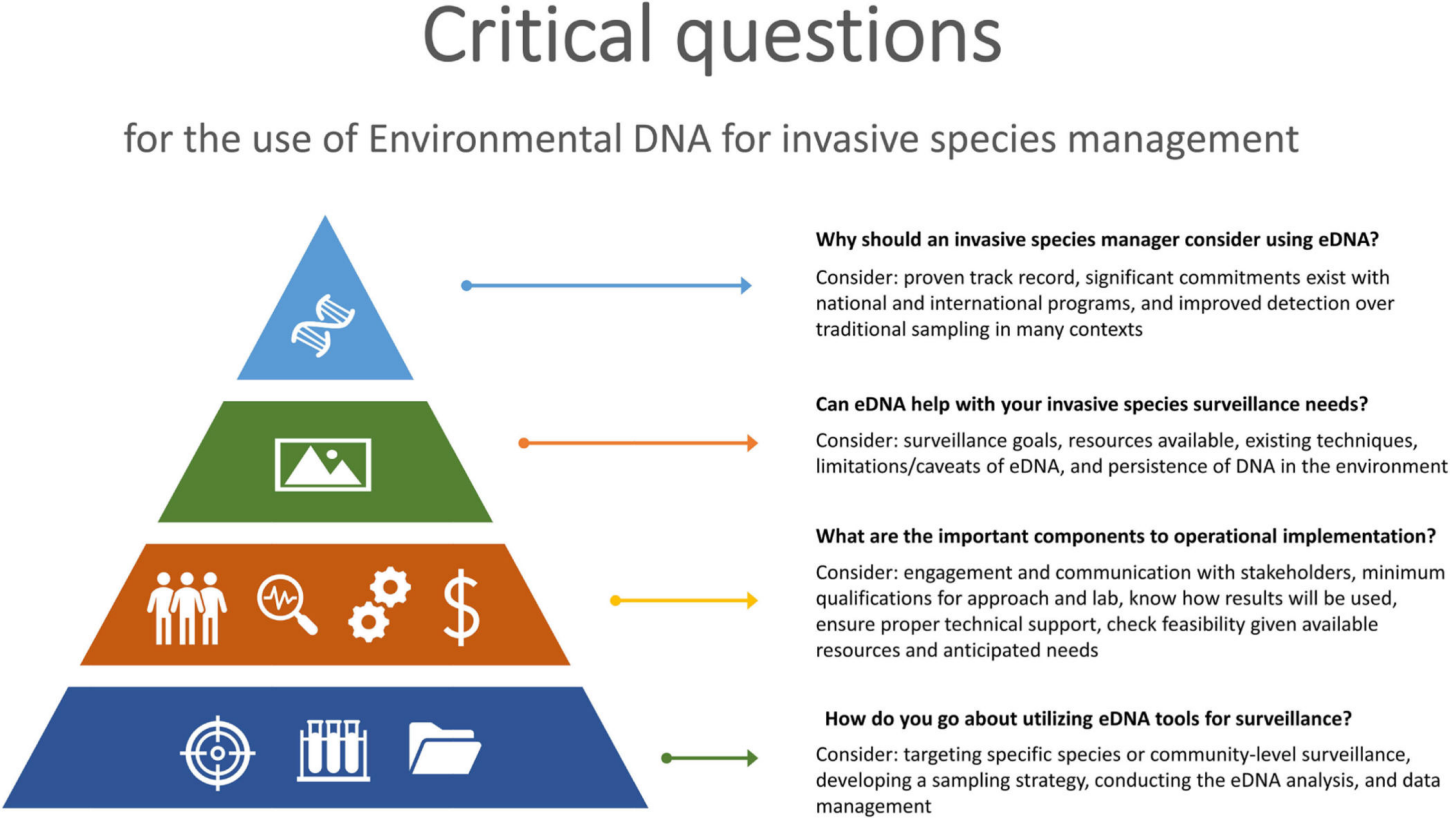
Critical question for the use of Environmental DNA for invasive species management.

We address these questions in this paper. We cover topics directed toward managers as well as practitioners working with eDNA techniques. Section II provides background on the utility of eDNA in invasive species management and points to various examples of its use across federal and international programs (question 1). Section III provides information on deciding whether eDNA is useful for a given application (questions 2). The implementation section (IV) describes practical considerations when using eDNA as a tool in invasive species management surveillance (question 3). The technical section (V) gives a high-level overview of the steps necessary for eDNA analysis (question 4). Figure 1 provides a graphic overview of the content and guidance covered in this paper. The conclusion section provides a summary of the paper and presents a flow diagram to capture the decision making and technical considerations highlighted in this paper.

## II. Background: Proven effectiveness and current programs related to the use of eDNA for invasive species surveillance

Considering there is extensive research on eDNA and its operational use, our focus in this section is an overview of research and resources that provide examples that are most relevant to invasive species management. Targets for detection of invasive species in aquatic environments using eDNA include groups as varied as vertebrates (e.g. North American bullfrog: *Lithobates catesbeianus* [Shaw, 1802], northern pike *Esox lucius* Linnaeus, 1758, silver carp *Hypophthalmichthys nobilis* [Richardson, 1845], black carp *Mylopharyngodon piceus* [Richardson, 1846]) (Ficetola et al. 2008; Jerde et al. 2013; Dunker et al. 2016; Jerde 2019; Stepien et al. 2019), aquatic mollusks and arthropods (e.g. quagga and zebra mussels *Dreissena bugensis* [Andrusov, 1897] and *D. polymorpha* [Pallas, 1771], rusty crayfish *Faxonius rusticus* [Girard, 1852]) (Dougherty et al. 2016; Amberg et al. 2019; Marshall and Stepien 2019; Sepulveda et al. 2019) and various plants (Newton et al. 2016). In marine systems, eDNA techniques are used to detect fouling communities (Westfall et al. 2020) and invasive species in ships’ ballast water (Gerhard and Gunsch 2019). Environmental DNA also has been used to detect terrestrial and semi-aquatic invasive species including the Burmese python (*Python bivittatus* [Kuhl, 1820]) (Piaggio et al. 2014), feral swine (*Sus scrofa* Linnaeus, 1758) (Williams et al. 2017), brown marmorated stink bug (*Halyomorpha halys* [Stål, 1855]) (Valentin et al. 2018), and spotted lanternfly (*Lycorma delicatula* [White, 1845]) (Valentin et al. 2020). As part of their Regional Aquatic Invasive Species Monitoring Strategy (USFS 2020a), the Pacific Northwest Region of the USDA Forest Service is employing eDNA to detect aquatic invasive species in environments that they do not regularly access with trained observers. These broad examples demonstrate the feasibility of using eDNA sampling to identify and detect invasive species within complex environments.

Environmental DNA sampling is non- or minimally-intrusive and often non-destructive. This, together with its specificity and broad contextual application makes the approach attractive as an invasive species detection tool (Kamenova et al. 2017; Hinlo et al. 2018; Martinez et al. 2020; Sepulveda et al. 2020a). Because most eDNA methods utilize polymerase chain reaction (PCR) of relatively short DNA fragments (generally < 200 nucleotides), they are sensitive enough to detect DNA at extremely low concentrations. Use of eDNA makes it possible to detect and identify invasive species effectively and to a rigorous standard (Frewin et al. 2015; Sepulveda et al. 2020a) and allows detection even when only a few specimens are present in the environment sampled and none have been captured or seen (Lyal and Miller 2020). Furthermore, specific taxonomic identification is facilitated through molecular techniques applied to eDNA. The field is rapidly evolving with continuous improvement in accuracy and at lower costs (Wilcox et al. 2016; Sepulveda et al. 2018; Pochardt et al. 2020; Thomas et al. 2020). As a further benefit, eDNA samples may be archived to be available for retrospective analysis for eDNA from other taxa. For example, samples from single-species surveys have been later used to screen for native mussels (Dysthe et al. 2018), amphibians (Franklin et al. 2019), and invasive fishes (Wilcox et al. 2018b, 2020).

While not necessarily explicitly directed toward invasive species management, multiple federal programs within the US have been instrumental in supporting research and technological advances in the use of eDNA. Table 1 provides a list of eDNA programs relevant to invasive species management in the US. These programs are motivated by the objectives inherent to the mission of each agency. Outside of the US, several nations and international efforts have been dedicated to pursuing the development and implementation of eDNA methods for various biomonitoring initiatives. Some of these are listed in Table 2. The tables are not an exhaustive list of federal and international eDNA programs. Rather, they are meant to show that there are numerous ongoing US federal agency, interagency, and international activities involving eDNA, with at least some components of that work relevant to the use of eDNA for invasive species management. The initiatives listed in Tables 1 and 2 represent investment and insight that can help inform further use of eDNA for invasive species management.

Details for each element of Tables 1 and 2 are provided in the Supplementary material Appendix 1.

While novel and exciting possibilities are offered through eDNA, with considerable investments in the related programs listed in Tables 1 and 2, there are still important caveats to consider. The following section describes some preliminary considerations for deciding on the use of eDNA for invasive species surveillance.

## III. Initial considerations: Deciding if eDNA is a useful tool in the context of specific invasive species management objectives

In considering the use of eDNA for invasive species surveillance, a critical first step is to carefully outline surveillance objectives. The context of the invasive species management objectives should articulate: the species of concern, the habitat of interest and the spatial extent of inference for the surveillance, the risks presented by the targeted species in that spatial extent, and the range of feasible management options. With respect to invasive species management, eDNA can be employed in a number of ways, including early and first detections of novel introduced species (Larson et al. 2020), tracking the spread of an introduced species (Dunker et al. 2016), or monitoring for survivors of eradication efforts (Carim et al. 2019). Criteria for useful tools may vary depending on these goals. For instance, challenges posed by detection uncertainty may be heightened when attempting early detection of a novel incursion and may be of less concern when seeking to establish range limits of a known invasion. In addition, it is important to establish how eDNA tools can be incorporated into the existing surveillance toolkit and used together with already employed methods. For example, is eDNA meant to provide the primary means of detection, leading directly to management action? Or is eDNA envisioned as an early screening approach, with positive detections triggering subsequent, more intensive monitoring using additional survey tools? Considerations of these objectives help to direct the eDNA methods to meet the manager’s requirements for surveillance sensitivity and specificity. Some of these questions are addressed, in part, in the following sections. However, to address unique needs inherent to invasive species management, these and similar deliberations are often best addressed through context-specific dialog between managers and eDNA practitioners.

An important early decision is if the eDNA surveillance objectives focus on 1) a targeted approach aimed at detecting a single species or genus or 2) a broader community assessment where multiple taxa of interest are characterized, known as eDNA metabarcoding. Here we provide an overview of community assessment methods, but specific considerations for its use in invasive species surveillance are beyond the scope of this paper. Although both approaches represent robust detection options, often outcompeting traditional survey methods, selecting a suitable method requires understanding trade-offs in sensitivity and specificity. A targeted eDNA approach uses taxon-specific primers and can be effective for identifying or monitoring elusive or rare species and is fitting for mapping the distribution for a particular invader of interest (Bylemans et al. 2019). Targeted (that is, species-, or genus- specific) approaches are highly sensitive in detecting targeted species (Hunter et al. 2017; Klymus et al. 2015; Marshall and Stepien 2019). However, a targeted eDNA approach is by definition limited to a specific “target” and is not suitable for the detection of unanticipated, or yet to be discovered, invasive species. eDNA metabarcoding provides an alternative option that can characterize multiple taxa, provide information about community diversity, and is appropriate to detect new or unanticipated invaders (Simmons et al. 2015; Snyder et al. 2020). One established flaw, however, is primer bias that may preferentially amplify (“select”) eDNA from particular taxa within a mixed community. As such, the eDNA metabarcoding approach may be less reliable in detecting any one specific species or taxon when a general marker is employed. For less common species or for broadly distributed species with substantial interpopulation genetic differences, there may be relatively low representation or diversity of DNA sequences in publicly available databases, such that important sequence variants are not accounted for in primer design, and detection rates of these invasive species or particular invasive species populations could be unexpectedly weak. This can be rectified by carefully considering community constituencies, phylogenetics, and species’ population genetics, to the extent possible, and obtaining representative samples and generating in-house sequence libraries as needed. Further, preservation and archival of extracted eDNA, samples, or filters may allow valuable back-tracing of the presence and tracking of invasive species that were present in a sample but unknown. With either approach, prior knowledge of a species’ phylogeography and invasion history is extremely useful (Parsons et al. 2018; Marshall and Stepien 2019). Community surveillance, using metabarcoding (Deiner et al. 2017) or multiplexed qPCR (Guan et al. 2019), can provide more information about the mix of species in the ecosystem but may be less specific or reliable at detecting any one specific species if a general marker is employed. Currently, metabarcoding is less applicable for regular invasive species monitoring, although this approach may be useful for detection of unanticipated incursions (Simmons et al. 2015; Snyder et al. 2020). What follows is guidance specific to the objective of using eDNA for surveillance of targeted species.

When pursuing targeted eDNA surveillance, it is useful to determine if known capabilities exist for the species of interest, or for related species. Existing studies may provide valuable information on ranges of DNA marker sensitivity and specificity (do certain markers/assays outperform others in side-by-side comparisons?; e.g. Sepulveda et al. 2020d), and may offer important insights in how and when to best sample the environment for that particular taxa. In the absence of such studies, general knowledge may be useful in evaluating the likely utility of eDNA for the target of interest. Knowledge pertaining to the ecology of the species (or genus) is particularly useful. This is because behavior and environmental condition may influence the rate at which DNA is shed into the environment (Pilliod et al. 2013) and may influence the fate and transport of eDNA in the system being monitored. A great deal of work has been done to understand how environmental variables (temperature, water quality, hydrology, etc.) influence the persistence and distribution of eDNA in the environment (Shogren et al. 2017).

For any given species and habitat, it is useful to understand the nuances related to both the risk of it remaining undetected (Meyers et al. 2020) and the careful interpretation of eDNA analyses in light of those risks. Both false negative and false positive errors are usual in any monitoring program (although technical solutions exist to minimize both, details are given below), and planning for allocation of limited resources should anticipate the costs associated with such errors. These considerations may also help to shape managers’ tolerance for different types of monitoring error, which in turn will help establish criteria for sensitivity and specificity of monitoring tools.

Recognizing the nuances of biology and environmental context when using eDNA for surveillance is important. While eDNA is extremely sensitive, the method can still result in false negatives, where results fail to detect target DNA when the target is present (e.g. when the species is in low abundance, Xia et al. 2018). Conversely, the methods can also result in false positives due to contamination or lack of specificity (Sepulveda et al. 2020b; Ficetola et al. 2016). Finally, and perhaps most uniquely challenging to eDNA methods, there is the possibility of detecting target DNA when the organism is not actually present (e.g. DNA coming from upstream) or there is not a living or viable population (e.g. DNA from a single carcass or from waste products of birds) (Merkes et al. 2014; Dunker et al. 2016). Although these can pose challenges for inferring distributions of underlying populations, it is important to consider the potential value of these types of detections as early warning signals. Knowing that there is target DNA entering a system—even if currently not associated with live organisms— may be the smoke indicating a potentially avoidable fire. These nuances are addressed in sections IV and V.

How eDNA surveillance will fit within the larger context of the invasive species and resource management plans, decisions, and actions is also important. The risk of invasive species going undetected can be used to inform a cost/benefit analysis of the surveillance efforts and inform the value of those efforts. Within any analysis of resource allocation, it is useful to establish the management options available for the species and habitat. There is higher value in detections that can trigger quick and effective actions. Detections that do not have associated meaningful actions are of little practical value. Investments in eDNA techniques should be done in a way that are proportional to the risk the species presents to the ecosystem and the availability and feasibility of effective actions.

Whether it is derived from a quantitative cost/benefit analysis or simply constrained by practical considerations, it is important to establish the resources (financial, personnel, and facilities) available for the surveillance. Using eDNA techniques will require allocation of time, budget, and access to the appropriate technology. Any dedication of resources to eDNA analysis should be done in light of the relative availability and efficacy of traditional or current monitoring tools and the trade-off for balancing limited resources between those tools and eDNA techniques. It is also useful to estimate the relative costs associated with doing nothing, as well as the costs associated with actions taken in response to error. All of the complexities listed in this section suggest that application of structured decision making (Keeney 2020; Smith 2020) could help with deciding on whether or not to employ eDNA techniques.

## IV. Implementation and practical considerations

Although eDNA has shown power to outperform some other invasive species monitoring tools (Valentini et al. 2016; Deiner et al. 2017; Boussarie et al. 2018), given the caveats listed above there are some important practical considerations when implementing eDNA methods. Establishing the surveillance objectives and risk tolerance up front can inform the decisions of which specific eDNA approach(es) to be employed (explained below), the minimum/required level of qualifications for the eDNA laboratory (referred to as “lab” throughout), validation of techniques to be used, and how results will be interpreted to inform actions. Due to the highly technical (and continually advancing) nature of eDNA methods and accompanying bioinformatic processing, the entire process, from initial planning to use of results, should include guidance from experienced eDNA practitioners or close adherence to robust guidance documentation (Darling 2019).

### Engagement and communication with stakeholders

As described above, important caveats, uncertainties, and unknowns are associated with eDNA results, and missteps in early adoption of eDNA tools combined with imperfect communication can lead to distrust of eDNA by managers (Amberg et al. 2015; Jerde 2019) and stakeholders. Herein “stakeholders” refers to the broader community beyond the managers and eDNA practitioners to include those impacted by the invasive species and related management actions. However, eDNA has been determined to be a viable, stand-alone method for rigorous decision making under the law using the Daubert standard, which evaluates reliability of scientific evidence in US Federal Courts (Sepulveda et al. 2020a). Nevertheless, eDNA results require careful use by managers since it can be difficult to determine whether or not eDNA detections are indicative of species presence, much less a viable population.

Project and communication plans jointly developed by managers and eDNA practitioners are an effective tool for averting missteps and misunderstandings. Such plans can build trust among managers, eDNA practitioners, and stakeholders. They can also ensure transparent decision making. These plans can build on the surveillance objectives, risk, and context articulated in the previous section. Furthermore, these plans can clearly identify the relevant decision makers, the eDNA sampling and analytical methods, limitations of these methods, criteria for scoring samples and sites as positive detections, and actions that will be taken if positive detection thresholds have been met. Importantly, plans must establish clear roles, responsibilities, and expectations among all parties and explicitly state how and when information will flow among the parties to ensure that there are no surprises. Project and communication plans should be developed before a study commences, but they should also be considered as living documents since eDNA sampling and analysis can be an iterative process. A useful example of a project and communication plan is the US Fish and Wildlife Service (USFWS) Quality Assurance Project Plan (QAPP) for eDNA Monitoring of Bighead and Silver Carps within the Great Lake Restoration Initiative eDNA monitoring (Table 1 and Appendix 1) (Woldt et al. 2019). This plan not only lays out specifics for the eDNA monitoring and communications, but it is also updated (approximately) annually and then approved by all parties. Detailed documentation is provided to describe why updates have occurred.

### Minimum qualifications for eDNA methods and lab

Multiple guidance documents now exist to inform minimum quality control standards in the field and lab, including Goldberg et al. (2016) “Critical considerations for the application of environmental DNA methods to detect aquatic species”, Thalinger et al.’s (2020) “A validation scale to determine the readiness of environmental DNA assays for routine species monitoring”, the USFWS QAPP (Woldt et al. 2019), the US Geological Survey (USGS) eDNA Nonindigenous Aquatic Species database submission criteria (USGS 2020), and the US Forest Service eDNA Atlas database submission criteria (Young et al. 2020). Quality control and assurance specifics will vary by project. More detailed information on quality control is given in section V. The quality control standards that are ultimately selected for the project should be jointly decided by all parties, especially the relevant managers and eDNA practitioners. Standards should align with the managers’ objectives and risk tolerance and should be thoroughly documented in the project and communication plans.

Labs that are accredited by recognized entities, such as the International Organization for Standardization (OECD 1997), are becoming more common in other countries. In the US, few academic, agency, or private labs have pursued accreditation. Consequently, the US lacks a nationally recognized and consistent approach to identify capable labs with successful quality assurance and quality control track records. However, the US-based web site “eDNA Resources” provides a list of commercial labs advertising eDNA sample processing services as well as US governmental labs processing eDNA samples (Washington State University 2020). The site also contains a document with “Considerations for selecting a lab partner”.

### Plan for how results will inform actions

Positive eDNA results should be interpreted as strong evidence (Sepulveda et al. 2020a) but do not conclusively establish the presence of a live occurrence of the species, much less a viable population of that species. Building on the decision to utilize eDNA and the inherent nuances of the biological interpretation of eDNA results, it can be helpful to explicitly agree to and document what managers will do with the information once available. Prior to the initiation of sampling, decision makers working with biologists (subject matter experts) and eDNA practitioners can identify specifically which eDNA results (e.g. from what locations, at what time, with what amount of replication or independent reproducibility) will trigger actions, how much certainty is required before these actions are triggered, and what course of actions will follow non-detection(s). For example, in the invasive carp eDNA monitoring program, the USFWS QAPP outlines that positive eDNA samples should undergo additional investigation, including subsequent, more intensive molecular and non-molecular monitoring to locate fish populations. These decisions should link manager objectives to alternative action choices, provide the information available to evaluate each alternative choice, and reflect management’s core-values. Here too, structured decision-making approaches (Smith 2020) could be used to work though these decisions.

Each practical consideration has associated costs. Once the engagement and communication strategies are established, the required technical capacity is known, and a plan outlined for how results will be used to inform actions, it is worth revisiting the resources available. It is important to understand what resources are available for the effort and establish the feasibility of the preceding implementation plans relative to the available resources. If the anticipated costs exceed the available resources, one or more of these practical considerations can be revisited and revised.

## V. Technical considerations

It may seem difficult to implement an eDNA monitoring or surveillance effort, given the myriad of critical considerations that occur at each step. However, several publications have provided detailed reviews of critical considerations that facilitate effective implementation of an eDNA program or effort (Goldberg et al. 2016; Stoeckle et al. 2018; Jeunen et al. 2019; Harper et al. 2019; Baillie et al. 2019). The following fairly high-level overview draws from the published literature and specifically aims to provide a general understanding of the process in the context of invasive species surveillance. This overview provides foundational information related to technical steps in conducting eDNA analysis for invasive species detection and/or surveillance.

### Assays

In most cases, an eDNA genetic assay is based on polymerase chain reaction (PCR) in which primers are used to amplify a locus (i.e., region of the genome) of interest possessing a unique sequence that is diagnostic of the focal taxa/taxon. Often, quantitative PCR (qPCR) assays incorporate a hydrolysis probe (e.g. TaqMan^™^) which produces a fluorescent signal when the target sequence is amplified. Loop-mediated isothermal amplification (LAMP) assays include four primers that bind to six portions of a locus. Colloquially, both the eDNA assay and the target locus may sometimes be referred to as an “eDNA marker.” Designing and testing (or validating) an assay is one of the most critical steps for eDNA approaches. It requires a great deal of care and often can be the most costly and time consuming step in starting an eDNA surveillance program. A list of *some* currently available assays can be found on the eDNA Resources website (Washington State University 2020).

The development of assays for novel invasive species requires three basic steps including: design and comparison to known DNA sequences representing nontarget organisms (*in silico* validation), laboratory tests with DNA extracted from related and/or sympatric species of the same type (*in vitro* validation), and testing of the assays in field habitats (*in situ*) where the target taxon is known to occur as well as in others where it is known to be absent. Standardized approaches and metrics exist for characterizing the performance of eDNA assays, such as limits of detection (LOD) and limits of quantitation (LOQ), which reflect the sensitivity of assays to low levels of target eDNA (Klymus et al. 2020). In the case of qPCR, there are well established standards for assay performance and reporting (Minimum Information for publication of Quantitative real-time PCR Experiments, MIQE) (Huggett 2020). Targeted assays may be designed that are specific to populations, species, or taxa of organisms.

If there is an eDNA assay available for the species of concern, managers can either work with the original laboratory that developed the eDNA assay or identify another laboratory that can implement the established assay. However, for an assay to be considered, it should be tested in the geographic region of interest against related species that the managers will likely encounter at their field sites. The manager can work with the lab to conduct additional tests to validate the assay for the field sites of interest. If there is no eDNA assay for the target species, a lab will need to develop an assay, which requires additional cost and effort to collect organismal samples and lab work to establish the assay.

### Develop a sampling strategy

The specific objectives of the surveillance will influence the creation of an effective sampling design for the target species and its associated environmental system (Goldberg et al. 2016; Wilcox et al. 2018a; Zinger et al. 2019). Because temporal, spatial, and seasonal variability in species distribution, behavior, and abundance will impact eDNA detection probability (Jerde et al. 2019; Harrison et al. 2019; Baillie et al. 2019), there are some important questions related to sampling design across time and space:

- What is the spatial and temporal distribution of the sampling locations?
- How should seasonality be considered to inform frequency and/or timing of samples?
- Will replicates be used to increase accuracy in light of weak signals?
- What is the cost per sample?
- Will there need to be trade-offs between sampling costs and accuracy requirements?

These questions should be considered against the surveillance goals and management objectives, with technical input from those familiar with the eDNA methods to be used, the species being considered, and the habitat where the surveillance will occur. There are further sampling considerations specific to eDNA techniques that should be assessed by the lab conducting the analysis. The lab should be able to specify the number of samples it can process in the time needed to meet the surveillance objectives. In addition, the lab’s quality assurance protocol (see the “Minimum qualifications for eDNA methods and lab” subsection above) will influence the sampling strategy.

Use of a pilot study can often inform the sampling strategy, even when an eDNA assay has been employed elsewhere for the same target species. A novel field site will introduce unique biotic challenges and DNA sequence diversity that may affect detection efficacy (Barnes and Turner 2016). A statistical analysis of pilot data and biotic characteristics of the field site can inform occupancy modeling which can be used to estimate occurrence and detection probabilities and thereby account for imperfect detection (Hunter et al. 2015). Pilot data analysis can also guide sample sizes and laboratory technical replicates required to obtain a desired probability of detection (Erickson et al. 2017; Hunter et al. 2017; Davis et al. 2018; Doi et al. 2019). A pilot study can also inform practical considerations related to the DNA capture methods (e.g. how fast does a filter clog?) and logistical constraints (e.g. getting to the collection sites). Finally, a pilot study can help confirm the feasibility of the proposed controls, degradation rates, and quality assurance measures (see following subsections).

### Conducting the eDNA analysis

#### Experimental and physical controls and validation levels

Positive and negative control samples are essential to improve interpretation of results and limit ambiguous findings. Both negative and positive experimental controls are needed in the field, during DNA concentration (filtration, centrifugation, etc.), and isolation and in the PCR plate (Snyder and Stepien 2020). Guidelines exist for the best practices of eDNA capture and isolation (Goldberg et al. 2016; Deiner et al. 2017; Taberlet et al. 2018; Stoeckle et al. 2018; Jeunen et al. 2019; Klymus et al. 2020; Minamoto et al. 2020; Shu et al. 2020). The lab utilized for the eDNA surveillance should be familiar with the most recent guidelines.

Positive field control samples from waterbodies or terrestrial field sites with independently established positive sightings of the target species can also be used to ensure that the assay and experimental protocols function efficiently in the field setting. Pilot studies under field conditions are necessary to assess the performance (sensitivity and specificity) of the assay that was developed and optimized under laboratory conditions. For example, the level of environmental compounds that inhibit the PCR (e.g. humic, tannic acid) may interfere with the ability of the assay to successfully detect the presence of eDNA and limit detection (Hunter et al. 2019; McKee et al. 2015). Internal positive controls (IPCs) should be applied to test that the reagents and PCR protocol are working effectively, even in the absence of target eDNA. Further, an assay may detect related species in field samples (i.e., false positives) that were not available for testing in the lab. To ensure the protocol and assay are not producing false positive results, negative field control samples should be collected from sites where the target species has never been recorded and is believed to not occur. In addition to classical positive and negative controls (as described above), a transportation control is also useful to assess whether contamination might occur while samples are transported from the field (e.g. in coolers) to the lab.

To reduce the likelihood of cross-contamination, minimum physical distances and specific controls are necessary in both field and lab spaces. These include the use of standardized and routine decontamination of all field and lab equipment using strong bleach solutions for extended periods. Note: This bleach must be thoroughly washed and completely removed from equipment before re-use as it can inhibit PCR and degrade DNA in newly acquired samples. It is additionally recommended that different stages of eDNA processing (i.e. concentrating, isolation, PCR) be completed in physically separate spaces with controlled airflow circulation, using equipment dedicated to the specific task/eDNA step. As pipettors can readily become contaminated, filter tips are critical to reduce the likelihood of eDNA or PCR cross-contamination throughout lab procedures.

Quality assurance and control measures during the acquisition of eDNA data and metadata should be completed as critical steps in the workflow protocol (Woldt et al. 2019). Standardization of within laboratory protocols (with quality control measures) and protocols to routinely assess the accuracy of the recorded data and metadata must be put in place to limit compounding errors within a dataset. Within the eDNA community, there is convergence (e.g. Baillie et al. 2019) on a 5-level validation scale developed by Thalinger et al. (2020) to assess the general quality of the study. It was developed as a user-friendly tool to evaluate previously published assays for future research and routine monitoring, while also enabling appropriate interpretation of results. It also provides guidance on validation and reporting standards. Environmental DNA practitioners can use this basic validation scale to determine if published assays are appropriate for application to their specific monitoring objectives.

#### eDNA capture and concentration

Most of eDNA capture and concentration work has been focused on aquatic systems and those are our focus here. However, soil, air and biological materials also are widely used for eDNA capture. eDNA collection methods vary but can include sampling from shorelines using buckets (Minamoto et al. 2020), at depth using Niskin bottles, or from aboard ships using various pumping devices (Hansen et al. 2008; Costello et al. 2017; Westfall et al. 2017; Minamoto et al. 2020). More recently, autonomous vehicles and systems have been deployed for sampling marine and aquatic systems at extreme depths or in river systems at stream gages (Yamahara et al. 2019; Sepulveda et al. 2020c).

Pumping water through a filter is the most common approach for eDNA capture in aquatic systems (Laramie et al. 2015). Vacuum or peristaltic pumps are commonly used for direct eDNA collection and concentration (Laramie et al. 2015; Goldberg et al. 2016) with both useful in either the field or lab. Alternatively, water samples can be centrifuged to concentrate cellular material. This method may be especially useful in turbid systems that clog filters (Snyder et al. 2020). Recent developments in eDNA capture method includes the use of filter housings (compatible with any suction pump) which are comprised of a biodegradable, hydrophilic material that functions to automatically preserve captured eDNA via desiccation (Thomas et al. 2019). Finally, filtered samples can be maintained on ice or stored at ambient temperature using a buffer, with evidence to suggest that buffers can maintain sample integrity for up to 56 days (Williams et al. 2016). The lab should have personnel with the technical expertise to work with the managers to recommend the best collection, filtering/concentration, and transport methods. However, since the actual work happens outside of the lab, collaboration between managers and eDNA practitioners can help establish the most efficient arrangements of personnel and resources to carry out the work. Here proper procedures, protocol, and oversight should be established among all personnel involved.

#### eDNA extraction

There are multiple methods of eDNA extraction (i.e., isolation and purification) from water samples and there is no single approach that is best for all study ecosystems or target species (Deiner et al. 2015; Jeunen et al. 2019). There are many commercially available kits (e.g. Qiagen DNeasy Kit™) and chemical methods (e.g. phenol/chloroform extractions) designed to isolate and purify DNA. Testing of various isolation methods, perhaps as part of initial pilot studies, is often an important step in determining the optimal method for a given study system (Deiner et al. 2015; Goldberg et al. 2016; Williams et al. 2017; Kumar et al. 2020; Shu et al. 2020).

#### eDNA amplification and quantification

The presence of target eDNA is assessed using polymerase chain reaction (PCR) and associated instrumentation/platforms. Some platforms, specifically, quantitative PCR (qPCR), and/or droplet digital PCR (ddPCR) (Doi et al. 2015; Goldberg et al. 2016; Klymus et al. 2020), can also provide the capacity to quantify amplified target DNA, which can provide estimates of relative abundance between sites. Also, hydrolysis probe-based qPCR and ddPCR (e.g. TaqMan™) provide added confidence that the detected eDNA indeed corresponds to the target locus within the target taxon (Wilcox et al. 2013). Quantitative conventional probe-based qPCR is currently the most commonly utilized eDNA amplification platform (Goldberg et al. 2016; Klymus et al. 2020) for single-species detection (for technical details see Taberlet et al. 2018). Although a newer technology, ddPCR, holds promise for increased eDNA utilization as it is less affected by environmental inhibitors (Doi et al. 2015; Hunter et al. 2017, 2018; Baker et al. 2018) and has been shown, in some cases, to outperform qPCR (Doi et al. 2015; Hunter et al. 2017).

Single-species targeted eDNA assays primarily reveal presence/absence of the species in the environment (Goldberg et al. 2016). However, sensitive and accurate assays and quantification platforms (i.e. qPCR) can allow for robust estimates of species density or biomass in a given habitat based on previously-determined correlations between calculated eDNA concentrations and taxon numbers or biomass per volume of aquatic habitat (Takahara et al. 2012; Chambert et al. 2018). The quantitation of eDNA amplification through qPCR or estimation of eDNA molecules per microliter from ddPCR has been used to estimate relative abundance of a species, although further research is needed to establish the accuracy of abundance or biomass estimates (Doi et al. 2015).

Accurate external standards are critical for quantification of qPCR assays. Quantification in ddPCR is considered absolute and does not rely on external standards. DNA analysis is performed in multiple wells using the same DNA extract template material and PCR reagents, with each replicate being referred to as a technical replicate or PCR replicate. Multiple replicates are run per sample because, when the concentration of target eDNA in the sample is low, there is a chance that replicates may lack target DNA even if the DNA is present in the sample (Sepulveda et al. 2020a). The more replicates that are analyzed per sample, the higher the probability that target DNA is present in at least one replicate. Running multiple replicates per sample not only improves the estimate of detection probabilities, but also helps estimate precision and strength of evidence. Some labs recommend 5 or 8 replicates (Klymus et al. 2020). The best number to use will depend on the assay, environmental covariates, and detection probabilities. Detection models can be applied to pilot data to help determine the number of PCR replicates and samples in order to achieve an identified detection threshold.

Amplification and quantification of eDNA and the number of sample replicates within the field and lab fall within the purview of the eDNA professional. However, these data will ultimately fall within the context of the plan for how results will inform action, described above. Therefore, clear communication and a plan a priori for how an actionable positive will be determined, based on the number of replicates (e.g. 1 positive out of 24 replicates is considered enough or not enough to implement management action), is critical between the eDNA experts and the managers.

### Metadata and data management life cycles

There is a growing convergence among those using scientific data to adhere to a concise and measurable set of principles known as FAIR: Findable, Accessible, Interoperable, Reusable (Wilkinson et al. 2016). These principles act as guidelines to enhance the application of data for other researchers and management activities, enhance the ability of machines to automatically find and use the data, and support its reuse by individuals for temporal, spatial or synthetic scientific analysis. Building on FAIR, there has been a more recent call for additional purpose-oriented principles known as CARE: Collective Benefit, Authority to Control, Responsibility, and Ethics. The CARE principles originated within the context of indigenous data governance (Carroll et al. 2020; GIDA 2019). The CARE principles complement the FAIR principles; encouraging open data movements to consider both people and purpose in their advocacy and pursuits. Both sets of principles are important for knowledge sharing on eDNA collection, analysis, management agencies that will enable the wider stakeholder community to improve the uptake of eDNA techniques.

The creation, validation, storage, and sharing of eDNA data under FAIR principles ensures the data are reliable, accessible, and in a format that allows for optimal impact and use by larger audiences, including managers, eDNA practitioners, and stakeholders. The creation and validation of eDNA data may occur in numerous labs but long-term storage and sharing often falls to a few agencies and databases. Carefully incorporating eDNA surveys into invasive species management requires access to reports documenting the analysis described in this section (V) as well as access to bioinformatic analysis and resulting data (Coble et al. 2018). Storing eDNA data on publicly accessible databases improves data access and engagement with managers or other practitioners, and ultimately supports data-driven decisions. Because various sources create eDNA data, it is essential to record metadata and track the history of long-term data sets. Metadata (including descriptors of collection methods) should be documented to enable analysis of combined data sets accumulated from discrete but related experiments. Metadata should accompany eDNA data as they are stored and shared to inform future surveillance work or application of similar surveillance in similar habitats and/or species.

It is important to integrate eDNA data across local, regional and national levels. This ensures that information sharing and/or efforts to control invasive species are not limited by geographic or agency boundaries. Invasive species surveillance in one area can support the use of eDNA and information transfer to other applications by sharing expertise, data, technologies and techniques and by working with labs in the development of standardized field, laboratory, and analysis protocols. A federally sponsored clearinghouse, curated and maintained to provide a single resource for up to date information, including peer-reviewed publications, agency reports and grey literature (e.g. relevant websites) could help in this regard.

Federal agencies should follow the federal metadata standard, FGDC (FGDC-STD-001–1998). However, no established standards currently exist that are specific to eDNA metadata. The USGS is actively working to remedy this and establish such standards. This work suggests that all metadata should provide: creator’s contact information; geographic locations; abbreviations, units, or codes used in the dataset; instrument and protocol information; experimental design; and version information. Data and metadata should follow the FAIR guiding principles (Wilkinson et al. 2016). (See the eDNA community standards created by Ferrante et al. *in prep*.).

An effort is underway to integrate eDNA data into the USGS Nonnative Aquatic Species (NAS) database (Table 1 and Appendix 1), providing a vehicle for efficient and rapid eDNA data sharing among local, state and federal agencies. Visual detections of invasive species are currently shared among groups using this database, and alerts are sent to subscribed managers and stakeholders. These data will be FAIR to allow for knowledge sharing across various audiences to improve uptake of scientific information. One example of collaboration across federal and state agencies is the Burmese python (*P. bivittatus*) eDNA tracking program. This program assesses the distribution of Burmese pythons across many jurisdictions throughout peninsular Florida. In this example, active coordination is ongoing among the US National Park Service, USFWS, and state agencies to ensure a common understanding of its distribution and productive and efficient management actions.

Proper metadata and sample archiving can extend the use of the sample into the future, to potentially include analyses involving additional taxa. Archived eDNA samples could provide a powerful tool for assessing species distribution through time, including tracking rates and extents of invasion spread. Archived eDNA samples could also be useful for evaluating advancements in eDNA technology, to compare and contrast results using new vs. old molecular tools. This sort of repurposing of eDNA samples has already become an integral part of the eDNAtlas database (Young et al. 2020) where samples from single-species surveys have been later used to screen for native mussels (Dysthe et al. 2018), amphibians (Franklin et al. 2019), and invasive fishes (Wilcox et al. 2018b, 2020). Effective repurposing relies on easy access to a long-term archival system while protecting samples from degradation and contamination. It is also important that managers and eDNA practitioners clearly communicate their expectations for long-term sample archival and potential re-analysis to ensure objectives are successfully met. Factors to consider before samples could be repurposed include garnering usage permission from the original collector/group, and understanding original eDNA sampling (specific habitat, season), collection method (filter material, pore size, volume filtered, etc.), extraction method, and storage method.

## VI. Summary and conclusions

**Figure 2.**
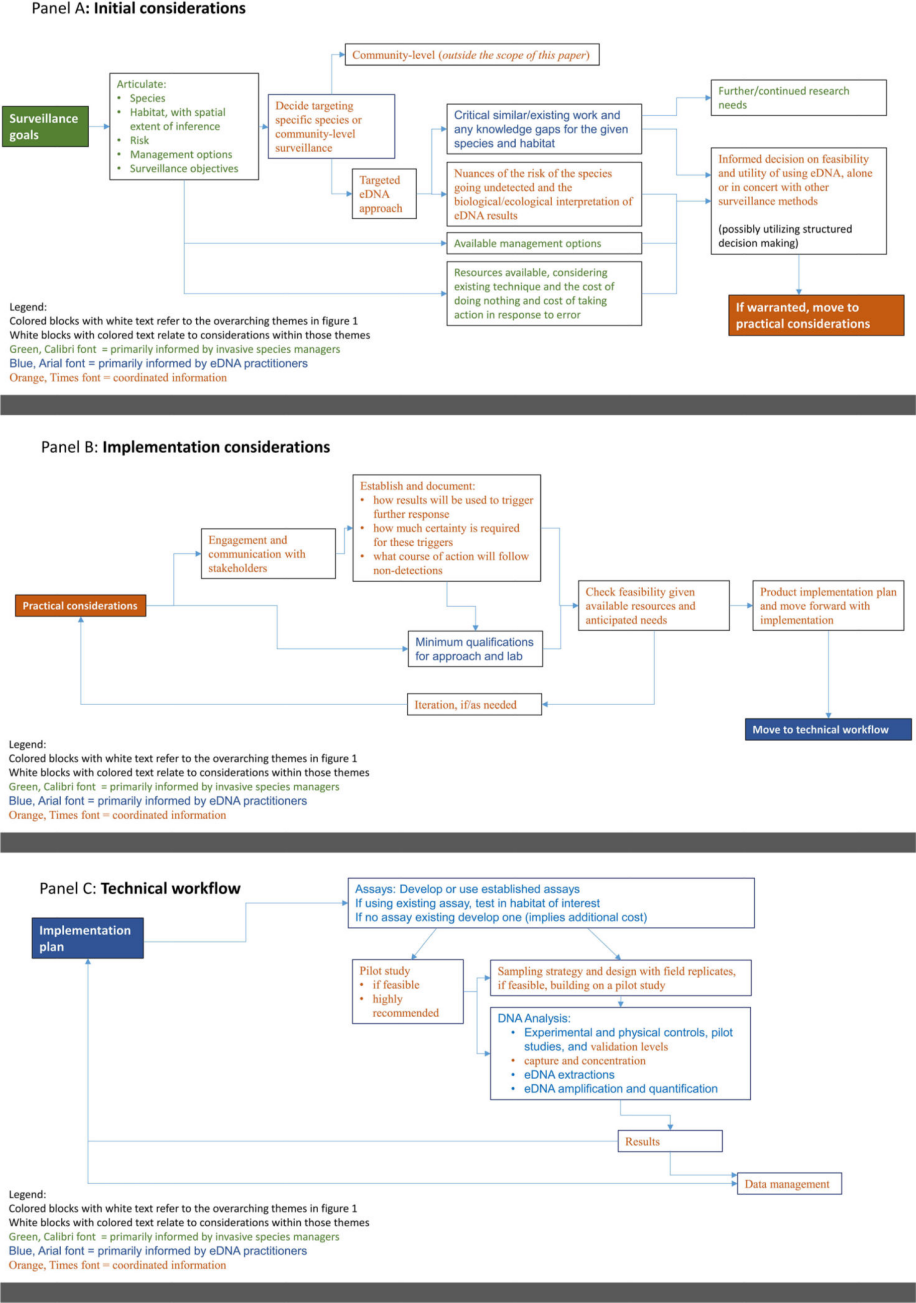
Workflow for the use of use of Environmental DNA for invasive species management, A: Initial considerations, B: Implementation considerations, and C: Technical considerations.

This paper has described key considerations for the use of eDNA within the context of invasive species management. Figure 1 provides an overview of these considerations. Figure 2 provides additional details on the processes involved with the three main phases: initial, implementation, and technical. The flow diagrams in Figure 2 visualize the general framework for utilizing eDNA for invasive species surveillance and the inter-relationships among the various components involved in using eDNA for invasive species surveillance. Context-specific issues will arise in each unique eDNA application, and in real-world situations emphasis may need to be placed on certain components, with less attention to others, resulting in slightly different flow or connections than outlined. However, in general, the framework should help managers in their deliberations regarding the use of eDNA for invasive species management by presenting a more holistic picture of what is involved.

Visualizing the workflow of eDNA for monitoring invasive species into different steps also allows for identification of where more specific plans, guidance, and/or protocols can be applied to help standardize and streamline its usage. Initial considerations can establish how the technology is suitable for management needs, surveillance goals, targets, risk tolerance, and other possible surveillance approaches. It could also detail management options available if/when the species is detected, as well as the implications of taking no action. Implementation considerations would cover the necessary arrangements with labs, partnerships and communications with other stakeholders, documentation of the planned surveillance process, and what actions will be triggered by eDNA results. Finally, technical considerations would include development or securing the required assays, pilot study(ies) to validate techniques, collection/sampling protocols, eDNA analysis and lab standards, and data management. It is also important to note the connections and feedback across the different steps as, for example, getting the final eDNA results is not the last step but rather leads back to the plans articulated in the implementation phase.

Recent work in translational ecology is motivated to drive outcomes that directly serve the needs of managers. This approach deliberately extends research beyond theory or opportunistic applications to address complex issues through interdisciplinary team approaches and integrated scientist-practitioner partnerships. Continued sharing of information and data from assays to protocols to lessons learned is a critical component of advancing and strengthening these linkages. Overall, this team approach is meant to both help shape use-driven, actionable science and foster higher levels of trust and commitment that are critical for long-term, sustained engagement among partners (Enquist et al. 2017).

Figure 2 attempts to distinguish which group plays the primary role in each step of the deliberations, implementation, and technical aspects of using eDNA for invasive species surveillance. As indicated in the legend, each element in the workflow is coded to indicate if that element is informed primarily by managers, eDNA practitioners, or jointly between the two communities. Articulating these roles helps emphasize the importance of using interdisciplinary scientist-manager partnerships.

This paper describes how eDNA can be used for invasive species surveillance. But just knowing it can be an effective tool is “the tip of the iceberg” (as indicated in Figure 1). There are important, substantial factors and challenges to consider, with valuable discussion needed among parties involved to develop an effective, readily usable, and interpretable eDNA survey/plan. The ultimate goal of this paper is to further assist managers in deciding if, when, and how to use eDNA for surveillance and, if it is used, how to follow an approach where results will be accepted by managers and by stakeholders with a clear understanding of the strengths and limitations of the method.

## Supplementary Material

Supplement1

## Figures and Tables

**Table 1.** US Environmental DNA programs with relevance to Invasive Species Management.

US Federal Programs	Agency	Relevance to invasive species	Citation

Aquatic Nuisance Species Task Force	Multiple plus non-federal partners	ANSTF focuses on invasive species; the Early Detection Rapid Response subcommittee as well as several regional panel working groups are working collaboratively on eDNA issues	Aquatic Nuisance Species Task Force 2020
eDNA Atlas: National Genomics Center for Wildlife and Fish Conservation	US Department of Agriculture Forest Service	Open-access database that provides spatial information on eDNA sampling detection/non-detection results for freshwater species in the United States	Young et al. 2020; USFS 2020b
eDNA Resources	Non-Federal but funded through the Department of Defense	A collection of information on using eDNA methods for the conservation and management of aquatic ecosystems; including the document “Guidelines for Selecting a Lab for Processing”	Washington State University 2020
Government Environmental eDNA Working Group	Multiple	Many members of the GeDWG are working on the application of eDNA to invasive species surveillance	Ferrante et al. 2020
Great Lakes Restoration Initiative eDNA monitoring	Multiple	The longest standing application of eDNA used for invasive species coordination and regional operational surveillance in the United States; providing an example of how eDNA can be implemented on a broad scale, across multiple jurisdictions to aid and inform invasive species detection and monitoring	Great Lakes Interagency Taskforce 2020
Intelligence Advanced Research Projects Activity: Detection approaches related to marine and coastal biosecurity	Department of Defense, Office of the Director of National Intelligence	Indication of IARPA’s interest in advanced methods including the use of eDNA; some of which could be directed toward invasive species surveillance	Office of the Director of National Intelligence 2006
Marine Biodiversity Observation Network (MBON) eDNA and ‘Omics coordination	Multiple US agencies plus international partners	Marine invasive species are one of the main threats being considered by this initiative	US MBON 2020
National Oceanic and Atmospheric Administration ‘Omics strategy and implementation	US Department of Commerce, National Oceanic and Atmospheric Administration	Indication of NOAA’s commitment to ‘omics which includes eDNA; with applications to invasive species explicitly mentioned under goal 2	NOAA 2019
US Fish and Wildlife training on eDNA	US Fish and Wildlife Service via the National Conservation Training Center	Focused on the use of eDNA’s in the management of plants and animals in general	Patterson 2020
US Geological Survey Nonindigenous Aquatic Species Database	US Geological Survey	The system is focused on nonindigenous species and expanding to include environmental DNA data	USGS 2020

**Table 2.** Environmental DNA programs with relevance to Invasive Species Management, international or outside the US.

Relevant programs outside the US	Country	Relevance to invasive species	Citation

DNAqua Net	European Union	Convenes a group of researchers across disciplines with the task to identify goldstandard genomic tools and novel eco- genomic indices and metrics for routine application for biodiversity assessments and biomonitoring of European water bodies.	Leese et al. 2016
Defra Centre of Excellence for DNA Methods	United Kingdom	Seeks progress on implementation of eDNA approaches including non-native species detections; aligned with Scottish DNA hub and the UK DNA network	Nelson et al. 2018
Guidance on the Use of Targeted Environmental DNA (eDNA) Analysis for the Management of Aquatic Invasive Species and Species at Risk Canada	Fisheries and Oceans	Guidance on eDNA to support decision making on aquatic species and ecosystems, considers both aquatic invasive species and species at risk	Abbott et al. 2021; Baillie et al. 2019
EcoDNA: a research group focusing on the application of environmental DNA technology for biodiversity conservation in Australia and the Asia-Pacific region	Led by University of Canberra, Australia	The mission, to provide advanced methods for species monitoring, includes work on invasive species	University of Canberra 2020
North Pacific Marine Science Organization	Canada, Japan, China, the Republic of Korea, Russia and US	Advisory Panel on Marine Non-Indigenous Species (AP-NIS) considering, in part, the application of eDNA for identifying invasive species in marine environments	PICES 2020
Arctic Invasive Alien Species (ARIAS) Strategy	Arctic countries (countries include Canada, Finland, Iceland, Norway, Sweden, Russia and US)	Action plan implementation is now leveraging eDNA tools to monitor invasive species	Arctic Council 2020
Pathway to Increase Standards and Competency of eDNA Surveys (PISCeS)	Canada	Advancing collaboration and standardization efforts in the field of eDNA	Loeza-Quintana et al. 2020
The Atlas of Living Australia	Australia	The Atlas of Living Australia includes invasive species records	CSIRO 2020
The eDNA Society	Japan	Standardized protocols applicable to all taxa, including invasive species	The Environmental DNA Society 2020
