# Supplementary material for: Strategic considerations for invasive species managers in the utilization of environmental DNA (eDNA): steps for incorporating this powerful surveillance tool
Source: Manag Biol Invasion. Author manuscript; Available in PMC 2022 Jul 9. (PMC8958948; doi:10.3391/mbi.2021.12.3.15)
Supplement: Supplement1 [file NIHMS1732071-supplement-Supplement1.pdf]

Supplemental material

Appendix 1. A summary of United States and international environmental DNA programs with relevance to invasive species management, as listed in Table 1 of the main paper.

US Federal programs

Aquatic Nuisance Species Task Force

In 1990, Congress established the Aquatic Nuisance Species Task Force (ANS Task Force) with the passage of the Nonindigenous Aquatic Nuisance Prevention and Control Act (NANPCA), which was reauthorized with the passage of the National Invasive Species Act (NISA) in 1996 (collectively, the Act) (US Department of Interior 1990). The Act charges the ANS Task Force with the responsibility of developing and implementing a program for waters of the United States to prevent the introduction and dispersal of ANS, to monitor, control, and study such species; and to disseminate related information. The ANS Task Force is co-chaired by the US Fish and Wildlife Service (USFWS) and National Oceanic and Atmospheric Administration and consists of 13 Federal agency representatives and 13 ex-officio representatives. These members work in conjunction with six regional panels and issue-specific committees to coordinate efforts among agencies as well as efforts of the private sector and other North American interests. Within the ANS Task Force structure, the Early Detection Rapid Response subcommittee as well as several regional panel working groups are working collaboratively on eDNA issues, including communicating the appropriate uses and limitations of eDNA methods to federal, state, tribal, and local agencies and the private sector.

The Aquatic eDNAtlas Project

The eDNAtlas is an open-access database that provides spatial information on eDNA sampling detection/non-detection results for freshwater species in the United States. Partnering with the National Genomics Center for Wildlife and Fish Conservation – a science collaborative within the Rocky Mountain Research Station of the US Forest Service – environmental samples are crowd-sourced from numerous natural resource agencies and non-governmental organizations that use a standardized field sampling protocol. At the time of writing, this database contains over 20,000 data points including thousands of sites and dozens of species and is updated annually. The database is currently piloting an expansion to incorporate data generated by multiple genetics laboratories.

eDNA Resources

The eDNA Resources site was created with funding from the Department of Defense (DoD) Environmental Security Technology Certification Program (ESTCP) and DoD Legacy Resource Management Program. It is currently managed by Washington State University (WSU) with a mix of content that was created by WSU for the site as well as eDNA resources contributed by the eDNA research community. The site provides introductory material on the use of eDNA and related reference materials, as well as information on implementation.

Government Environmental DNA Working Group

The US Federal Government eDNA Working Group (GeDWG) was established to make connections, share information, and catalyze collaboration between US federal employees on work related to environmental DNA (eDNA) methods, tools, data handling, and resources. The group is a community of practice among federal employees working with eDNA. Membership is open to any interested US Federal employee or eDNA practitioners working on federal projects. The group organizes an annual workshop on eDNA technical exchange. Invasive species topics comprise a significant portion of the presentations and considerations at these workshops and many members of the GeDWG are working on the application of eDNA to invasive species surveillance.

Great Lake Restoration Initiative eDNA monitoring

This project is an example of how eDNA can be implemented on a broad scale, across multiple jurisdictions to aid and inform invasive species detection and monitoring. The Great Lakes eDNA program is the longest standing application of eDNA used for invasive species coordination and regional operational surveillance in the United States. While it is an example of an ongoing operational program, it is useful to note that it continues to evolve. With the assistance of USFWS Fish and Wildlife Conservation Offices, the Whitney Genetics Laboratory (WGL) seeks to continually test and refine the collection, processing, analysis, and interpretation of eDNA samples and results. The WGL is dedicated to ensuring the best methods are being used to improve detection probabilities and to effectively communicate results to partners so they can make informed management decisions. WGL staff work closely with other Service Technology Centers across the nation and collaborates with US Geological Survey experts to validate new eDNA tools and to conduct occupancy models to better inform eDNA sampling for invasive Carp. The Quality Assurance Project Plan (QAPP) for eDNA Monitoring of Bighead and Silver Carp is an essential part of the ongoing program. As mentioned in the main paper, the QAPP not only provides examples of planning and documenting how eDNA results are disseminated to partners in a Communications Plan, it describes the improvements made to the program on a yearly basis. Some notable recent improvements include streamlining and automation of the entire eDNA sample

workflow process, from collection through analysis. For example, the ArcGIS Collector App is used to collect field data digitally and upload it to a database live, saving time and eliminating transcription errors; ArcGIS Survey 123 is used to generate and transfer chain-of-custody forms between the field crew and laboratory staff to ensure samples are tracked and handled properly throughout the process; and R and RMarkdown are used to automatically process eDNA detection data and generate standardized reports. The QAPP is updated annually and is publicly available through the USFWS eDNA webpage. Bighead and Silver Carp eDNA detections are publicly posted through the USFWS's Great Lakes Bighead and Silver Carp eDNA Monitoring Dashboard and on the USFWS's Midwest Region Fisheries Program eDNA website. The eDNA website showcases static tables and maps with detection information while the dashboard displays eDNA detections in an interactive map across multiple basins in a searchable format, allowing users to isolate detections for a specific location or time period.

Intelligence Advanced Research Projects Activity: Detection approaches related to marine and coastal biosecurity

In 2018 the Intelligence Advanced Research Projects Activity (IARPA) solicited information on detection approaches related to marine and coastal biosecurity. This Request for Information (RFI) was issued solely for information gathering and planning purposes; the RFI did not constitute a formal solicitation for proposals. As noted in that solicitation, methods of monitoring for AIS in coastal waterways and ballast water as well as on ship hulls exist, but current approaches are limited and time consuming. In addition, interpretation of complex biological data in ways that provide actionable information to federal, state, and local agencies can be difficult. Challenges with monitoring are compounded by geographic scale, the diversity of life found within these systems, the limited extent of marine reference data, and changes resulting from environmental conditions such as storms, microclimates, and seasonal changes. Development and advancement of capabilities for the detection of AIS is needed to enable early detection of risks to food, water, and economic resources. Through this solicitation, IARPA gathered information regarding robust, cost-effective, and standardizable tools for early detection of AIS in both marine and fresh-water environments, ports and marinas, ballast water, and on ship hulls. While there is no explicit commitment of funding related to this initial information-gathering stage, it does provide evidence of the IARPA's interest in advanced methods including the use of eDNA.

Marine Biodiversity Observation Network (MBON) eDNA and 'Omics coordination

The Marine Biodiversity Observation Network (MBON) is a global initiative composed of regional networks of scientists, resource managers, and end-users working to integrate data from existing long-term programs to better understand changes and connections between marine biodiversity and ecosystem

functions. Marine invasive species are one of the threats being considered by this initiative and their impact to marine biodiversity and native species abundance. The US MBON projects are integrating independent historical and current biology and ecosystem surveys with new observations, and expanding application of remote sensing methods, novel molecular technologies (including eDNA), traditional environmental research tools, and coordinated experiments. MBON projects have developed best practices for eDNA and demonstrated its utility for biological observing, identification of ecosystem indicators, and understanding biodiversity change. In addition to the development of molecular methods, MBON projects have advanced the means to collect samples for eDNA analysis using autonomous underwater vehicles. At the time of writing there were 15 ‘Omics and eDNA related publications from the MBON Projects.

National Oceanic and Atmospheric Administration ‘Omics strategy and implementation

’Omics refers to a suite of advanced methods used to analyze material such as DNA, RNA, proteins, or metabolites. NOAA’s vision, expressed through its ‘omics strategy is to integrate modern ‘omics technologies across the agency, transforming its approach to biological investigation and accelerating sustainable management of ecosystem resources for the benefit of people, communities, and economies. The overarching aim of the NOAA ‘Omics Strategy is to ensure investment in ‘omics technologies to meet mission priorities. The strategy contains five overarching goals. Goal #2 is to “Execute ‘omics research targeted to support and advance the American Blue Economy”. Under that goal there is Objective 2.1: to improve detecting and monitoring of harmful algal blooms, toxins, pathogens, and invasive species to protect health and coastal economies. Thus, through this national strategy, there is evidence of NOAA’s commitment to explore the use of eDNA as well as an explicit articulation to focus effort on invasive species.

US Fish and Wildlife training on eDNA

The USFWS’s National Conservation Training Center (NCTC) provides training courses on a wide variety of conservation topics. NCTC courses are primarily designed to serve the needs of USFWS personnel but courses are available to the wider conservation community. As part of the Emerging Topics in Conservation Science Workshop series, the NCTC developed a workshop on eDNA in 2020. The target audience for the workshop is resource professionals who work with issues where eDNA data have implications for the management of plant or animal populations, especially those in aquatic habitats. The offering is designed to provide resource professionals at both staff and management levels with the background and tools needed to evaluate issues where eDNA data play a role in the management of plants

and animals. Workshop topics include eDNA Best Management Practices, terminology, concepts, and case histories. Participants learn how to evaluate eDNA information in the scientific literature and explain how it relates to management decisions; evaluate project proposals and reports and identify the appropriate environmental data collection and analysis methods needed to answer questions of management concern; describe the benefits and limitations of applying eDNA methods to management issues; and develop and review appropriate study designs using the Best Management Practices and principles of eDNA.

US Geological Survey Nonindigenous Aquatic Species Database

Nonindigenous Aquatic Species (NAS) Database was established by the US Geological Survey as a central repository for spatially referenced biogeographic accounts of introduced aquatic species. The program provides scientific reports, online/real-time queries, spatial data sets, distribution maps, and general information. The data are made available for use by biologists, interagency groups, and the general public. The geographical coverage is the United States. The program is expanding the database to include eDNA data with the objective of consolidating its traditional specimen sightings with eDNA detection data and associated metadata into a single database. Thus, it will provide more complete distribution records and improve national early detection and rapid response capacities for aquatic invasive species.

Programs outside the US

Outside of the US, several nations have been aggressively pursuing the development and implementation of eDNA methods for various biomonitoring initiatives. Some have begun to incorporate these tools into decision making.

Australia

The Atlas of Living Australia – a government-run, open-access database of species distribution records – has recently incorporated eDNA data (www.ala.org.au/environmentaldna). The country hosts an active and productive eDNA research community including commercial (e.g. EnviroDNA; www.envirodna.com), academic (e.g. TrEnD; www.trendlab.com.au), and governmental (e.g. CSIRO; research.csiro.au/environomics) initiatives.

Canada

Canada’s Department of Fisheries and Oceans (DFO) has been promoting a number of eDNA research projects to support client needs in aquatic invasive species as well as species at risk, marine protected areas, fisheries, aquaculture, biodiversity assessments, etc. (Baillie et al. 2019). In addition, DFO supports a National eDNA Technical Working Group and has sponsored two national eDNA workshops. DFO is

also working with the Canadian Standards Association to initiate the development of Canadian standards for eDNA (Helbing and Hobbs 2019). In parallel with this, DFO developed science advice for aquatic invasive species and species at risk clients (initiated by the Canadian Federal and Provincial National Aquatic Invasive Species Committee) on minimum reporting standards, terminology and guidance on interpretation of data (Abbott et al. 2021). That guidance is likely to form the basis of a policy statement on the adoption and implementation of eDNA monitoring in Canada. DFO is also engaged on eDNA standardization and technical development with academic and industry partners through the Pathway to Increase Standards and Competency of eDNA Surveys (PISCeS) semi-annual conference and working group (Loeza-Quintana et al. 2020). A useful component to the Canadian guidance document is an accompanying reporting template. This template provides a tangible and practical way to help ensure the guidance is followed (Baillie et al. 2019).

European Union

Perhaps the most significant advances have been made in the European Union (EU) under the auspices of the DNAqua-Net EU Cost Action, a multidisciplinary network of researchers aiming to identify genomic tools for biomonitoring and general biodiversity assessments (Leese et al. 2016). Monitoring of aquatic ecosystems in Europe is generally conducted under the Water Framework Directive (WFD; 2000/60/EC) and the Marine Strategy Framework Directive (2008/56/EC), with each EU member state tasked with developing and applying assessment systems under these umbrellas. Many member states are now moving toward DNA-based assessment systems to complement classical field and lab methods already in use. In principle the EU committee overseeing ecological assessment as part of the WFD (ECOSTAT) is open to the uptake of new methods pending intercalibration of the novel methods. These approaches encompass both targeted eDNA monitoring as well as community metabarcoding. A guide on intercalibration and compliance checks exists.

More than a year ago, DNAqua-Net made an inventory of projects underway in Europe to develop DNA-based methods with the goal to implement them into standard monitoring and counted 40 independent projects. Many of these are parallel testing or validation studies conducted by individual countries, e.g. SCANDNAnet (Finland, Sweden, Iceland, Denmark, Norway), GeDNA (primarily Germany but also involving Switzerland and Austria), and SYNAQUA (France, Switzerland). Currently there is a formal eDNA working group within the European Standards Committee (CEN/TC230/WG28). Through DNAqua-Net in collaboration with the European Committee for Standardization (CEN) Standardization working group and ECOSTAT in the near future there is likely further central coordination of DNA-based bioassessments in the EU.

Japan

Japanese researchers established the *eDNA Society* in 2018 (Minamoto et al. 2020) (https://ednasociety.org/). A key product of the society has been a standardized protocol for eDNA sampling, analysis, and interpretation which has already been implemented for several academic and government projects within the country.

New Zealand

In New Zealand (NZ) much development of DNA-based tools for general biomonitoring has been driven by interest from and in coordination with oil and gas industry partners. These entities are interested in implementation of tools that can quickly and cost-effectively assess environmental change. A key to this is the possibility of collecting environmental samples that reliably yield quality DNA for future testing, so that baselines can be established even in retrospect. Standardization of sampling protocols and sample curation are therefore being prioritized. In addition, NZ Environmental Protection Authority has been adopting a “bottom-up” approach to incorporation of eDNA biomonitoring by conducting multiple citizen science projects with local and tribal entities. These projects are not designed so much to acquire actionable data, but rather to introduce those entities to the potential utility of eDNA approaches and to bring them on as partners in building more robust programs.

Recently, researchers at the Cawthron Institute completed a long-term project to assess the utility of metabarcoding approaches (focusing on microbial indicators but also incorporating eukaryotic indicators) for monitoring the integrity of marine benthic environments associated with salmon farming. Those environments have been traditionally monitored using an Enrichment Index based on both conventional indicators (benthic macroinvertebrates) and geochemistry, supported by the Board of Enquiry of the Environmental Protection Authority. Interest in incorporating DNA-based metrics was initiated through the Benthic Standards Working Group with input from stakeholders including industry, local communities, government (Ministry of Primary Industries and Regional Councils), and science providers (Cawthron Institute and the National Institute of Water and Atmospheric Research). The final outcome of

the government-funded project was the elaboration of a fully standardized protocol which will be applied unchanged during a ‘phase-in’ period of up to five years as part of regular compliance monitoring. It is anticipated that after this ‘phase-in’ period, DNA-based assessments will be sufficiently grounded (i.e., robust, consistent, and interpretable) for routine use and supported by environmental legislation in New Zealand.

United Kingdom

In the United Kingdom (UK) there are signs of increasing interest at the government level. Last year saw the launch of the Defra Centre of Excellence for DNA Methods, which seeks progress on implementation of eDNA approaches to achieve various government priorities, including non-native species and biodiversity assessments. That group is aligned with both the recently established Scottish DNA hub and the UK DNA network.

Progress toward implementation has already been achieved for a number of specific target groups, including non-native fishes and benthic invertebrate communities in both freshwater and marine systems. By far the most advanced is the great crested newt (*Triturus cristatus* [Laurenti, 1768]), a protected species in the UK under the Wildlife and Countryside Act, 1981 and a Priority Species under the UK Post-2010 Biodiversity Framework. Positive eDNA detections for *T. cristatus* are now accepted as legal basis for determining their presence in disputes over land development, so long as testing follows accepted protocols (Natural England 2015). Freshwater diatoms are another target group for which DNA-based monitoring approaches have been implemented. In 2017 the Environment Agency in England adopted a metabarcoding approach for ecological status assessment using diatoms in order to fulfil obligations under the WFD (Kelly et al. 2018). The DNA-based approach is meant to provide an alternative means for acquiring the same data on diatoms that have been traditionally collected via morphological approaches, a “molecular mirror” of the existing assessment method. Significant correlation between the molecular and conventional approaches was demonstrated and deemed sufficient to warrant adoption of the new tools (Kelly et al. 2018).

Intergovernmental Coordination

Since various countries have different environments and species that require different sampling standards and policies regarding invasive species, there are potential opportunities for lessons learned and shared methodologies for data collection and decision making. Intergovernmental bodies are exploring how eDNA is being used as a monitoring tool for invasive species and how best to use these tools for management and policy. The North Pacific Marine Science Organization (countries include Canada, Japan, China, the Republic of Korea, Russia and United States) through the Advisory Panel on Marine Non-Indigenous Species (AP-NIS) is scheduled to host a workshop related to discussing this topic

amongst the "North Pacific" countries at an upcoming meeting. Arctic countries (countries include Canada, Finland, Iceland, Norway, Sweden, Russia and United States) have developed the Arctic Invasive Alien Species (ARIAS) Strategy and Action Plan and through its implementation are now leveraging eDNA tools to monitor invasive species.

References

Abbott C, Coulson M, Gagné N, Lacoursière-Roussel A, Parent GJ, Bajno R, Dietrich C, May-McNally S (2021) Guidance on the Use of Targeted Environmental DNA (eDNA) Analysis for the Management of Aquatic Invasive Species and Species at Risk. DFO Can. Sci. Advis. Sec. Res. Doc. 2021/019. iv + 42 p, https://www.dfo-mpo.gc.ca/csas-sccs/Publications/ResDocs-DocRech/2021/2021_019-eng.pdf (accessed 7 April 2021)

Baillie SM, McGowan C, May-McNally S, Leggatt R, Sutherland BJG, Robinson S (2019) Environmental DNA and its applications to Fisheries and Oceans Canada: National needs and priorities. https://waves-vagues.dfo-mpo.gc.ca/Library/40803764.pdf (accessed 10 October 2020)

Helbing CC, Hobbs J (2019) Environmental DNA Standardization Needs for Fish and Wildlife Population Assessments and Monitoring. CSA Group, Toronto, Canada, 38 pp, https://www.csagroup.org/wp-content/uploads/CSA-Group-Research-Environmental-DNA.pdf

Kelly M, Boonham N, Juggins S, Kille P, Mann D, Pass D, Sapp M, Sato S, Glover R (2018) A DNA based diatom metabarcoding approach for Water Framework Directive classification of rivers. Environment Agency, Bristol, United Kingdom, 157 pp, https://assets.publishing.service.gov.uk/government/uploads/system/uploads/attachment_data/file/684493/A_DNA_based_metabarcoding_approach_to_assess_diatom_communities_in_rivers_-_report.pdf

Leese F, Altermatt F, Bouchez A, Ekrem T, Hering D, Meissner K, Mergen P, Pawlowski J, Piggott J, Rimet F, Steinke D, Taberlet P, … Zimmermann J (2016) DNAqua-Net: Developing new genetic tools for bioassessment and monitoring of aquatic ecosystems in Europe. *Research Ideas and Outcomes* 2: e11321, https://doi.org/10.3897/rio.2.e11321

Loeza-Quintana T, Abbott CL, Heath DD, Bernatchez L, Hanner RH (2020) Pathway to Increase Standards and Competency of eDNA Surveys (PISCeS)—Advancing collaboration and standardization efforts in the field of eDNA. *Environmental DNA* 2: 255–260, https://doi.org/10.1002/edn3.112

Minamoto T, Miya M, Sado T, Seino S, Doi H, Kondoh M, Nakamura K, Takahara T, Yamamoto S, Yamanaka H, Araki H, Iwasaki W, Kasai A, Masuda R, Uchii K (2020) An illustrated manual for environmental DNA research: Water sampling guidelines and experimental protocols. *Environmental DNA* 00: 1–6, https://doi.org/10.1002/edn3.121

Natural England (2015) Great crested newts: surveys and mitigation for development projects. United Kingdom, https://www.gov.uk/guidance/great-crested-newts-surveys-and-mitigation-for-development-projects

US Department of Interior (1990) USFWS. Nonindigenous Aquatic Nuisance Prevention and Control Act of 1990, Digest of Federal Resource Laws of Interest to the U.S. Fish and Wildlife Service. https://www.fws.gov/laws/lawsdigest/nonindi.html (accessed 20 October 2020)
